# PRC2 functions in development and congenital disorders

**DOI:** 10.1242/dev.181354

**Published:** 2019-10-01

**Authors:** Orla Deevy, Adrian P. Bracken

**Affiliations:** Smurfit Institute of Genetics, Trinity College Dublin, Dublin 2, Ireland

**Keywords:** PRC2, NSD1, DNMT3A, Weaver syndrome, Sotos syndrome, Tatton-Brown–Rahman syndrome

## Abstract

Polycomb repressive complex 2 (PRC2) is a conserved chromatin regulator that is responsible for the methylation of histone H3 lysine 27 (H3K27). PRC2 is essential for normal development and its loss of function thus results in a range of developmental phenotypes. Here, we review the latest advances in our understanding of mammalian PRC2 activity and present an updated summary of the phenotypes associated with its loss of function in mice. We then discuss recent studies that have highlighted regulatory interplay between the modifications laid down by PRC2 and other chromatin modifiers, including NSD1 and DNMT3A. Finally, we propose a model in which the dysregulation of these modifications at intergenic regions is a shared molecular feature of genetically distinct but highly phenotypically similar overgrowth syndromes in humans.

## Abstract (precis)

**Summary:** This Review discusses the molecular biology of PRC2, the developmental phenotypes associated with its loss of function in mice, and its crosstalk with other chromatin regulators in the context of human overgrowth syndromes.

## Introduction

Every multicellular organism begins life as a single cell that gives rise to the many functionally diverse cell types of the developing and adult organism. The specification of different cell types from this individual cell with a fixed genetic code depends on changing gene expression patterns, which can in turn be influenced by chromatin structure. Chromatin structure can be regulated by covalent modifications made to histone proteins or DNA, which are mediated by several different families of chromatin- and DNA-modifying enzymes (Soshnev et al., 2016).

Polycomb group (PcG) proteins are one such family of chromatin-modifying enzymes that function as repressors of gene expression, specifically of genes encoding key developmental regulators (Schuettengruber et al., 2017). PcG proteins function as part of multiprotein complexes that can be classed into two main types: Polycomb repressive complex (PRC) 1 and PRC2 (Bracken et al., 2019). PRC2 catalyses the addition of up to three methyl groups at histone H3 lysine 27 (H3K27me1/2/3) and can be further subdivided into two main forms: PRC2.1 and PRC2.2 (Bracken et al., 2019; Laugesen et al., 2019; van Mierlo et al., 2019). Similarly, PRC1 can be subdivided into canonical PRC1 (cPRC1) and non-canonical PRC1 (ncPRC1). cPRC1 ‘reads’ PRC2-mediated H3K27me3 and homodimerises, thereby promoting the physical compaction of chromatin (Kundu et al., 2017). In contrast, ncPRC1 is recruited to chromatin independently of H3K27me3 to deposit a single ubiquitin moiety on histone H2A lysine 119 (H2AK119ub), which is believed to contribute to the subsequent recruitment of PRC2.2 (Bracken et al., 2019; Laugesen et al., 2019; van Mierlo et al., 2019).

PcG proteins were first discovered in *Drosophila* as negative regulators of Hox gene expression during fly development, but are now known to be widely conserved across eukaryotes and to play a key role in lineage specification and cellular memory (Schuettengruber et al., 2017). Unsurprisingly, studies across several species have revealed that loss-of-function mutations in genes encoding Polycomb proteins can have deleterious and often lethal effects during development (Akasaka et al., 1996; Moazed and O'Farrell, 1992; San et al., 2016). A classic Polycomb mutant phenotype manifests as defective body plan patterning, typically marked by posterior homeotic transformations wherein anterior body structures display features of more posterior structures (Akasaka et al., 1996; Lewis, 1947; Slifer, 1942). The importance of PRC2 function during development is highlighted by the fact that homozygous mutations in the genes encoding each of its core components – EZH2, EED and SUZ12 – cause early embryonic lethality in mice (Table 1).

**Table 1.**
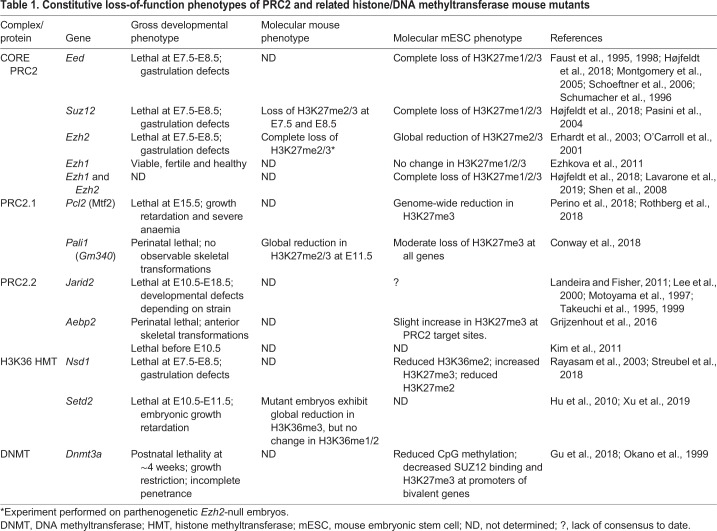
**Constitutive loss-of-function phenotypes of PRC2 and related histone/DNA methyltransferase mouse mutants**

In humans, heterozygous mutations in the *EZH2*, *EED* and *SUZ12* genes cause congenital overgrowth, often marked by features that are typical of those observed in Weaver syndrome (Table 2). For simplicity, we hereafter collectively refer to this group of disorders as being Weaver syndrome, a condition characterised by tall stature, a distinctive facial appearance and variable intellectual disability (Tatton-Brown et al., 2013). Two remarkably similar human overgrowth conditions, known as Sotos syndrome and Tatton-Brown–Rahman syndrome, are caused by heterozygous mutations in the genes encoding two other chromatin regulators, namely nuclear receptor-binding SET domain 1 (NSD1) and DNA (cytosine-5)-methyltransferase 3A (DNMT3A), respectively (Table 2), suggesting some level of functional interplay between PRC2, NSD1 and DNMT3A. Similar to Weaver syndrome, these conditions are marked by childhood overgrowth, dysmorphic facial features and learning disabilities (Okamoto et al., 2016; Tatton-Brown et al., 2005).

**Table 2.**
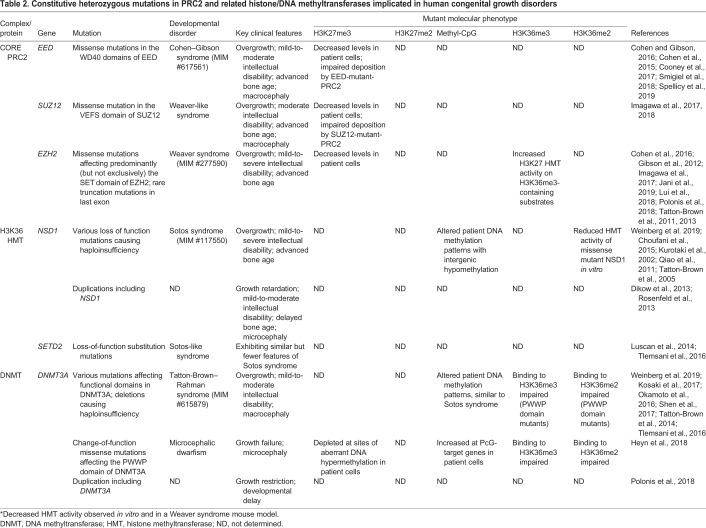
**Constitutive heterozygous mutations in PRC2 and related histone/DNA methyltransferases implicated in human congenital growth disorders**

In this Review, we discuss the latest advances in our knowledge of the molecular biology of PRC2 and present an updated summary of the developmental phenotypes associated with its loss of function in mice. We also describe recent molecular insights that are illuminating the regulatory interplay between the activities of PRC2, NSD1 and DNMT3A on chromatin. Finally, we propose a model in which a common feature of the above-named developmental disorders is misregulation of PRC2, NSD1 and DNMT3A function at intergenic chromatin, which in turn may account for their remarkable degree of phenotypic overlap.

## PRC2 composition in mammals

**Fig. 1.**
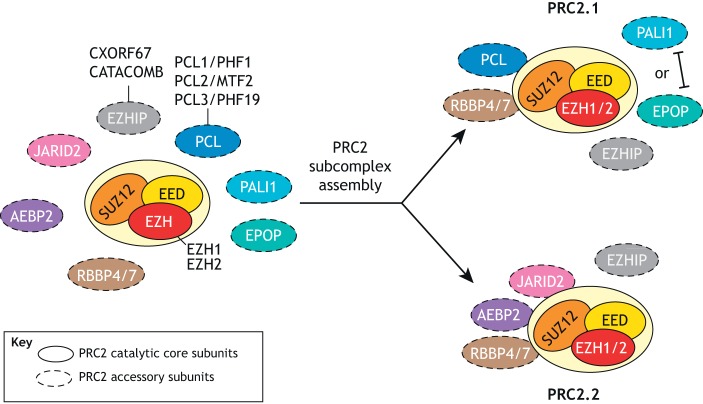
**Polycomb repressive complex 2 assembles into two subcomplexes.** Schematic of the subunits of mammalian Polycomb repressive complex 2 (PRC2), including both core and accessory subunits. The composition of the PRC2.1 and PRC2.2 subcomplexes are also depicted. Note that PALI1 and EPOP are mutually exclusive members of PRC2.1, whereas RBBP4/7 associates with both PRC2.1 and PRC2.2. EZHIP has also been reported to interact with both PRC2.1 and PRC2.2 but is expressed in a limited range of cell types. Paralogous subunits and alternative protein names are listed where applicable. Contacts shown between subunits are merely schematic and are not intended to reflect actual physical interactions.

Mammalian PRC2 consists of three core subunits (Fig. 1): SUZ12, EED and either the EZH2 or EZH1 histone methyltransferase (HMT) (Laugesen et al., 2019; Yu et al., 2019). These core PRC2 proteins associate in a 1:1:1 stoichiometry and catalyse all mono-, di- and tri-methylation of histone H3 lysine 27 (H3K27) through the SET domain of the EZH1/2 subunit (Højfeldt et al., 2018; Smits et al., 2013). This trimeric core of constitutive PRC2 components can associate with an expanding list of facultative or ‘accessory’ PRC2 components, including AEBP2, JARID2, PCL1/2/3 (PHF1/MTF2/PHF19), RBBP4/7 and the more recently identified EPOP, PALI and EZHIP proteins (Fig. 1) (Conway et al., 2018; Holoch and Margueron, 2017; Hübner et al., 2019; Jain et al., 2019; Ragazzini et al., 2019). Although these accessory components are not strictly essential for the formation of core PRC2, we are continuing to discover how they function to modulate its recruitment and enzymatic activity (Bracken et al., 2019; Laugesen et al., 2019).

As mentioned above, mammalian PRC2 exists in two mutually exclusive subtype assemblies – PRC2.1 and PRC2.2 – that are defined by their associations with specific accessory subunits (Fig. 1) (Alekseyenko et al., 2014; Grijzenhout et al., 2016; Hauri et al., 2016; Oliviero et al., 2016; Streubel et al., 2018). PRC2.1 contains one of the three paralogous Polycomb-like (PCL) proteins as well as either EPOP or PALI1 (Beringer et al., 2016; Conway et al., 2018; Liefke et al., 2016), whereas PRC2.2 is defined by the presence of the JARID2 and AEBP2 accessory proteins (Holoch and Margueron, 2017). *PALI2* is a paralogue of *PALI1*, the protein product of which is predicted to interact with PRC2 through a conserved ‘PIP’ domain (Conway et al., 2018). Like RBBP4/7, the most recently described accessory subunit, EZHIP, has been reported to interact with both PRC2.1 and PRC2.2, although it is not as widely expressed (Hübner et al., 2019; Jain et al., 2019; Piunti et al., 2019; Ragazzini et al., 2019). The discovery of these variant PRC2 assemblies poses a question that remains to be answered: what are their distinct functions, if any?

It is interesting to note that the subdivision of PRC2 into two main forms, containing either Pcl or Jarid2, is conserved in *Drosophila*, and that a homologue of AEBP2 (Jing) also exists in flies (Bracken et al., 2019; Herz et al., 2012; Nekrasov et al., 2007). It is therefore likely that any functional divergence between the two main PRC2 subtypes precedes the most recent common ancestor of flies and mammals, but has been expanded upon in the vertebrate lineage with the emergence of the PALI, EPOP and EZHIP accessory proteins.

The increase in the number of PRC2 proteins in mammals compared with fly is due to the occurrence of two whole-genome duplication events at the base of the vertebrate lineage (Dehal and Boore, 2005; Schuettengruber et al., 2017). For example, whereas *Drosophila* possess a single Polycomb-like protein (Pcl), mammalian genomes encode three such homologues (Brien et al., 2015). Furthermore, the EPOP, EZHIP and PALI1/2 PRC2.1 components do not exist in flies, but are instead specific to therians, eutherians and vertebrates, respectively (Beringer et al., 2016; Conway et al., 2018; Ragazzini et al., 2019). It will be interesting to assess whether the emergence of these additional PRC2 accessory proteins correlates with certain anatomical innovations during vertebrate evolution and/or certain tissue-specific functions not shared with our most recent common ancestor with *Drosophila*.

Should differential functions for PRC2.1 and PRC2.2 be identified, there is evidence to support the convergent evolution of functionally distinct PRC2 subcomplexes in plants. Although plants do not possess any known orthologues of the abovementioned PRC2 accessory proteins, they have multiple homologues of the core PRC2 components (Schuettengruber et al., 2017). *Arabidopsis*, for example, possesses three *SUZ12* homologues (*EMF2*, *VRN2*, *FIS2*) that define at least three different PRC2 subcomplexes that are known exhibit to divergent tissue- and/or developmental stage-specific functions (Derkacheva and Hennig, 2014). Interestingly, in mammals SUZ12 is the PRC2 subunit that mediates interactions between core PRC2 and accessory components, and therefore may be considered an independently evolved but analogous determinant of functional PRC2 subcomplex specificity (Laugesen et al., 2019).

## The molecular activities of PRC2 in mammalian cells

### Recruitment to chromatin

To date, the mechanisms by which PRC2 is recruited to chromatin have been less well-defined in mammals compared with *Drosophila*. However, clues as to how mammalian PRC2 is recruited to its target genes on chromatin are emerging through the study of its accessory proteins. Below, we provide an overview of reported PRC2 recruitment mechanisms in mammals. For further details, we refer readers to recent comprehensive reviews (Bracken et al., 2019; Laugesen et al., 2019; van Mierlo et al., 2019; Yu et al., 2019).

PRC2 binds to stretches of unmethylated CG-rich DNA, called ‘CpG islands’, at the promoters of inactive developmental genes in mammalian cells (Deaton and Bird, 2011; Ku et al., 2008; Lee et al., 2006; Riising et al., 2014). Its recruitment is facilitated, at least in part, by the Polycomb-like and JARID2 accessory components of the PRC2.1 and PRC2.2 complexes, respectively (Oksuz et al., 2018; Youmans et al., 2018). The Polycomb-like proteins contain a winged-helix domain that allows them to bind to unmethylated GC-rich DNA, whereas JARID2 is believed to recognise ncPRC1-mediated H2AK119ub at CpG islands via its ubiquitin interaction motif (UIM) (Blackledge et al., 2014; Choi et al., 2017; Cooper et al., 2016; Farcas et al., 2012; Li et al., 2017; Perino et al., 2018; Wu et al., 2013). Supporting this model, ncPRC1-mediated H2AK119ub1 is central to the role of JARID2 in promoting H3K27me3 during X-chromosome inactivation (Almeida et al., 2017; da Rocha et al., 2014). Furthermore, the loss of H2AK119ub1 in mouse embryonic stem cells (ESCs) lacking ncPRC1 function leads to a partial reduction in the levels of core PRC2 members and H3K27me3 at Polycomb target genes (Blackledge et al., 2019 preprint; Tamburri et al., 2019 preprint; Fursova et al., 2019; Scelfo et al., 2019). Recent studies have identified that PRC2 recruitment predominantly occurs via its targeting to unmethylated CpG islands, both directly by Polycomb-like proteins and indirectly by JARID2 via its association with ncPRC1-mediated H2AK119ub (Healy et al., 2019; Højfeldt et al. 2019).

It remains to be elucidated what, if any, contributions AEBP2, PALI1, EPOP or EZHIP make to PRC2 recruitment mechanisms. Unexpectedly, AEBP2 is reportedly capable of targeting PRC2 to methylated CpGs *in vitro* (Wang et al., 2017). This finding appears incongruent with several studies reporting an anti-correlation between PRC2 and methyl-CpG DNA genome wide (Bartke et al., 2010; King et al., 2016; Lynch et al., 2011). Therefore, it remains to be determined whether this methyl-CpG binding preference holds true *in vivo* and, if so, what functional contribution this might make to PRC2 action in cells.

### Enzymatic activity

PRC2-mediated H3K27 methylation can be catalysed by either EZH1 or EZH2. EZH2 is the more potent H3K27 methyltransferase and can fully compensate for EZH1; *Ezh2*-null cells show global reduction of H3K27me3 and H3K27me2, whereas *Ezh1*-null cells do not exhibit any reduction in H3K27 methylation levels (Table 1). Nevertheless, EZH1 can maintain normal levels of H3K27me1 in the absence of EZH2, and ablation of both EZH1 and EZH2 is required to abolish all H3K27 methylation (Højfeldt et al., 2018; Lavarone et al., 2019; Shen et al., 2008).

PRC2-mediated H3K27me3 has long been known to play a role in gene silencing (Laugesen et al., 2019; Margueron and Reinberg, 2011; Pengelly et al., 2013; Yu et al., 2019). It is broadly deposited across the gene bodies and flanking regions of transcriptionally silent developmental genes and leads to the recruitment of cPRC1, which initiates chromatin compaction and transcriptional repression (Bracken et al., 2019). The PRC2-mediated H3K27me2 modification is less well-characterised, despite being ubiquitously distributed across intergenic sites of the genome (Conway et al., 2015; Streubel et al., 2018). It has been proposed to function as a repressive ‘blanket’, possibly preventing the inappropriate activation of enhancers of alternative lineages (Conway et al., 2015; Ferrari et al., 2014; Lee et al., 2015). In this sense, H3K27me2 could be considered to represent the ‘default’ setting on chromatin. In contrast, the H3K27me1 modification is located along the bodies of actively transcribed genes, and correlates with the promotion of gene expression (Ferrari et al., 2014; Lee et al., 2015).

Almost all PRC2 accessory subunits enhance the HMT activity of the core complex *in vitro* (Laugesen et al., 2019). The one exception identified so far is the so-called enhancer of zeste inhibitory protein (EZHIP), which inhibits H3K27 methylation by binding the SET domain of EZH2 and preventing its methyltransferase activity (Hübner et al., 2019; Jain et al., 2019; Piunti et al., 2019; Ragazzini et al., 2019). Interestingly, whereas the loss of PALI1, JARID2 or Polycomb-like proteins in cells leads to reduced levels of H3K27me3, the loss or depletion of AEBP2 or EPOP results in an increase in H3K27me3 (Table 1) (Beringer et al., 2016; Conway et al., 2018; Grijzenhout et al., 2016; Højfeldt et al., 2018; Oksuz et al., 2018). Moreover, *Aebp2^−/−^* mice exhibit anterior transformation of the skeleton, as opposed to the classic Polycomb phenotype, i.e. posterior homeotic transformation (Grijzenhout et al., 2016). These paradoxical mutant phenotypes highlight that much remains to be done to understand the distinct and potentially subtle or context-dependent functions of different PRC2 accessory components. Some clues are emerging from studies of *Aebp2*-null mouse ESCs (Conway et al., 2018; Grijzenhout et al., 2016). These studies suggest that although AEBP2 likely does stimulate the HMT activity of PRC2.2 *in vivo*, its loss skews the balance of PRC2 subtypes towards the more catalytically active PRC2.1.

## PRC2 functions in mouse development

The requirement for PRC2 activity during mammalian embryogenesis is best exemplified by studies of germline loss-of-function mutations in *Ezh2*, *Eed* and *Suz12* in mice (Table 1). Loss-of-function mutants of these core PRC2 components invariably exhibit gastrulation defects and lethality around embryonic day (E)7.5-8.5, during early post implantation stages (Faust et al., 1995; O'Carroll et al., 2001; Pasini et al., 2004). Incidentally, loss-of-function mutations in the gene encoding the H3K36 histone methyltransferase NSD1 (discussed in detail later) also result in gastrulation defects and embryonic lethality at E7.5-8.5 (Rayasam et al., 2003). Death at this critical developmental stage reflects the essential functions of these chromatin regulators during early embryogenesis. Interestingly, although EZH1 is a core PRC2 subunit, *Ezh1* knockout mice have been reported as ‘viable, fertile, and healthy’ (Ezhkova et al., 2011), suggesting that EZH2 can compensate for its loss during development.

Loss-of-function mutants for accessory PRC2 subunits tend to exhibit more variable phenotypes, with lethality occurring in later embryonic, perinatal or early postnatal stages of development (Table 1) (Conway et al., 2018; Grijzenhout et al., 2016; Rothberg et al., 2018). However, it is important to bear in mind that this does not necessarily translate to a lesser importance for the PRC2 accessory components in development. Rather, it may indicate that a level of functional redundancy exists between PRC2.1 and PRC2.2. Consider that the loss of a core PRC2 component will render both forms of the complex completely non-functional, whereas the loss of an accessory PRC2 component should affect one subcomplex only and leave the other functionally intact. It is likely that the more subtle accessory mutant phenotypes are the result of skewing the normal balance of PRC2 subtypes, as opposed to the outright loss of PRC2 activity.

*Jarid2* mutant mice exhibit a range of phenotypes, the severity and age of onset of which appear to depend on the genetic background of the mice (Lee et al., 2000; Motoyama et al., 1997; Takeuchi et al., 1995, 1999). To date, all reported constitutive *Jarid2* loss-of-function mutations have been generated by gene trapping, and cause pre-natal lethality between E10.5 and E18.5 in mice (Table 1). Similarly, the phenotypes of the different reported *Pcl2*-null mice range from lethal at E15.5 to viable (Li et al., 2014; Rothberg et al., 2018; Wang et al., 2007). Potential sources of such phenotypic variation lie not only in the genetic background of the mice but also on the methods used to generate the null allele. For example, gene-trapping can cause different phenotypes depending on the vector insertion site (McClive et al., 1998; Olson et al., 1996). Additionally, gene-trap mutants can express tissue-specific, alternatively spliced forms of the gene in question, giving rise to hypomorphs rather than full knockouts. This phenomenon is exemplified by one strain of *Jarid2* gene-trap mutant mice, homozygotes of which retain leaky *Jarid2* expression in the nervous system (Lee et al., 2000). Alternative splicing has also been problematic with the *Pcl2* gene, which is expressed as multiple different isoforms (Li et al., 2014; Stanford et al., 2001). A more recent approach may have circumvented this issue by generating a *Pcl2* knockout mouse through a combination gene-trap and gene-targeting strategy (Rothberg et al., 2018). Accordingly, the resultant mice display the most severe and early-onset developmental phenotype, with none surviving past E15.5. Given the parallel roles of Polycomb-like proteins and JARID2 in directing the recruitment of PRC2.1 and PRC2.2 across the genome, it is entirely credible that *Pcl2* knockout mice should display a comparable knockout phenotype to their *Jarid2^−/−^* counterparts. Furthermore, it is reassuring to note that all prior reported *Pcl2* mutant mice display some degree of the classic Polycomb defect (posterior skeletal transformation) and/or postnatal lethality (Li et al., 2014; Wang et al., 2007).

A similar disparity exists between the two published *Aebp2* loss-of-function mouse mutant phenotypes, with lethality reported to occur either postnatally or at E10.5 (Grijzenhout et al., 2016; Kim et al., 2011). Both groups employed a gene-trap strategy to target *Aebp2*, suggesting that the phenotypic variation in this instance relates to the genetic background of the mice. What is most interesting to note is that one group observed an unexpected anterior homeotic transformation, or ‘Trithorax’ (Schuettengruber et al., 2017), phenotype in their *Aebp2*-gene-trapped mice (Grijzenhout et al., 2016). This has since been interpreted as resulting from a skew in the balance of PRC2 subtypes towards the more catalytically active PRC2.1 (Conway et al., 2018).

Setting aside the potential for functional redundancy between PRC2.1 and PRC2.2, the relatively delayed phenotypes of PRC2 accessory subunit mutants could indicate that their key functions are executed during organogenesis (E10-E14) and/or the foetal growth and development stages (E14 onwards), rather than during early embryogenesis. This is consistent with a model in which the core part of PRC2 is essential for the correct execution of cell fate specification during early embryogenesis, whereas the accessory PRC2 components come to the fore during later developmental stages to refine the activity of PRC2 and thereby give rise to progressively more differentiated and/or specialised cell types. An alternative interpretation could be that the consequences of the loss of PRC2 accessory proteins can be compensated for by the embryo, or the mother, up to that point in development. For example, despite the presumed persistence of some PRC2 activity via PRC2.2, PALI1-deficient embryos already show a reduction in global levels of H3K27me2/3 at E11.5, but do not exhibit lethality until the perinatal period (Conway et al., 2018). The functional consequences of these early molecular changes are tolerated by the embryos until birth, suggesting that: (1) compensatory mechanisms exist to overcome this reduction in PRC2 enzymatic activity; or (2) the physical manifestation of this reduction in H3K27me2/3 is not of functional consequence *in utero*. Another interpretation for the milder loss-of-function phenotypes of accessory PRC2 proteins could simply be that different accessory subunits are expressed in different tissues and at different stages during development, and that the penetrance and/or severity of the phenotype is rather a reflection of how essential the tissue(s) are for normal developmental progression. For example, a PRC2 accessory protein required for heart development would be expected to cause an earlier lethal phenotype than a subunit required for lung development. This is because a working circulatory system is essential for the embryo *in utero*, whereas independent breathing is not required until after birth. Therefore, to understand better the potential differential functions of PRC2 accessory proteins, it would be useful to generate a catalogue of their spatiotemporal expression patterns during embryonic development. Conditional loss-of-function mutant mice could then be generated to validate functionally their importance in particular lineages and/or at specific developmental stages.

As none of the PRC2 accessory subunit loss-of-function mutants phenocopies the loss of a core PRC2 protein, it can be inferred that no single PRC2 accessory subunit is required for the initial recruitment and function of core PRC2 during mouse embryogenesis. As such, it would be interesting to generate mice with loss of both JARID2 and Polycomb-like proteins to evaluate how this might compare with loss of core PRC2 activity.

## A role for imbalanced crosstalk between PRC2, NSD1 and DNMT3A in human developmental disorders

**Fig. 2.**
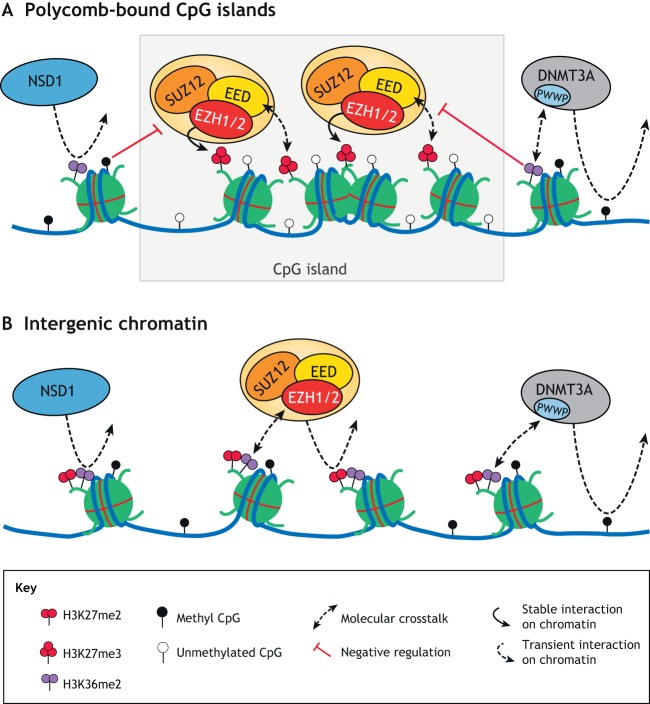
**The crosstalk between PRC2, NSD1 and DNMT3A on chromatin.** (A) Antagonistic interplay between NSD1-mediated H3K36me2, DNMT3A-mediated DNA methylation and PRC2-mediated H3K27me3 around Polycomb-bound CpG islands. Both DNA methylation and H3K36me2 are reported to antagonise the accumulation of PRC2-mediated H3K27me3. H3K27me3 allosterically activates PRC2. (B) Co-existence of PRC2-mediated H3K27me2, NSD1-mediated H3K36me2 and DNMT3A-mediated DNA methylation at intergenic chromatin. The crosstalk between these different chromatin regulators is illustrated by the ability of both DNMT3A and EZH2 to read the methylation status of H3K36.

Germline heterozygous mutations in the genes encoding core PRC2 members (*EZH2*, *EED* and *SUZ12*), *NSD1* and *DNMT3A* have been implicated in a triad of highly phenotypically related human developmental disorders: Weaver syndrome, Sotos syndrome and Tatton-Brown–Rahman syndrome, respectively (summarised in Table 2). As introduced earlier, NSD1 is a histone methyltransferase that catalyses the addition of up to two methyl groups on histone H3 lysine residue 36, and DNMT3A is one of two mammalian *de novo* DNA methyltransferases (Bennett et al., 2017; Rose and Klose, 2014). At the molecular level, PRC2, NSD1 and DNMT3A exhibit context-dependent functional interplay or ‘crosstalk’ on chromatin (Fig. 2). Deciphering this molecular crosstalk may prove key to understanding the molecular aetiology of the Weaver, Sotos and Tatton-Brown–Rahman syndromes. Below, we review recent insights into this crosstalk and summarise the phenotypes and genotypes of each of the related disorders in turn. Finally, we propose a model in which the overlapping phenotypes of these genetically distinct overgrowth disorders could be due to imbalances in the landscape of chromatin modifications at intergenic regions.

### Crosstalk between PRC2, NSD1 and DNMT3A

The activity of PRC2 on chromatin is subject to crosstalk from NSD1 and DNMT3A, which methylate H3K36 and CpG dinucleotides, respectively. Like H3K27, H3K36 can be modified by the addition of up to three methyl groups (Wagner and Carpenter, 2012). The NSD family of histone methyltransferases contains three members (NSD1-3) that catalyse the addition of up to two methyl groups on H3K36 (Wagner and Carpenter, 2012). In mammals, the histone methyltransferase SETD2 can then convert H3K36me2 to H3K36me3 (Edmunds et al., 2008). The ability of PRC2 to methylate H3K27 is influenced by the methylation state of H3K36 on the same histone such that its reaction rate is decreased on nucleosomes containing pre-existing di- or tri-methyl H3K36 modifications (Schmitges et al., 2011; Yuan et al., 2011; Zheng et al., 2012). In other words, the modification of H3K36 creates a less permissive environment for PRC2-mediated methylation at H3K27 (Fig. 2). Important insights into how this crosstalk could be mediated came from the recent identification of a binding pocket on EZH2 that reportedly acts as a molecular sensor to detect the methylation state at H3K36 (Jani et al., 2019). The data suggest that PRC2 is activated *in cis* by unmodified H3K36, but this activation effect diminishes in the presence of an increasingly more methylated H3K36 residue (Jani et al., 2019). This would be predicted to lead to an inverse correlation between the methylation states at H3K36 and H3K27 in cells. Supporting this, H3K27me1 colocalises with H3K36me3 along the bodies of actively transcribed genes, and H3K27me2 colocalises with H3K36me2, both being broadly deposited across the genome (Ferrari et al., 2014; Streubel et al., 2018). Interestingly, despite the fact that 30-50% of all histone H3 is dimethylated at H3K36, the function of this modification still lacks thorough characterisation (Rose and Klose, 2014). Some important clues into its function came from the discovery that reduced H3K36me2 in NSD1-depleted cells leads to a quantitative increase and qualitative expansion in H3K27me3 deposition, with concomitant decreases in H3K27me2 (Streubel et al., 2018). Furthermore, dimethylation of either H3K27 or H3K36 drastically reduces the rate of tri-methylation occurring on the alternate residue, suggesting that an equilibrium exists between these two modifications once established (Zheng et al., 2012). One could theorise that blankets of H3K36me2 and H3K27me2 exist across broad regions of the genome (particularly at intergenic sites, which lack H3K36me1/3 and H3K27me1/3) to together function as the ‘default’ setting on chromatin, limiting the potential for aberrant deposition of either activating H3K36me3 or repressive H3K27me3 marks (Fig. 2). In this model, decreases in the levels of intergenic H3K36me2 would shift the balance of PRC2-mediated methylations towards H3K27me3, at the expense of H3K27me2 (Streubel et al., 2018).

Molecular crosstalk also exists between H3K36 methylation and DNA methylation. Both di- and tri-methylation of H3K36 are known to recruit the *de novo* DNA methylases DNMT3A and DNMT3B to CpG-rich DNA via their PWWP (Pro-Trp-Trp-Pro) domains (Fig. 2) (Chen et al., 2004; Dhayalan et al., 2010; Ge et al., 2004). DNMT3B preferentially colocalises with H3K36me3 and methylates DNA along active gene bodies, a preference not shared with DNMT3A (Baubec et al., 2015; Weinberg et al., 2019). Like H3K36 di- and tri-methylation, DNA methylation is considered antagonistic to the deposition of PRC2-mediated H3K27me3 (Fig. 2) (Bartke et al., 2010; Reddington et al., 2013; Wu et al., 2010). Consistent with this, H3K27me3 levels are reported to increase globally in mouse ESCs completely lacking DNA methyltransferase activity (Hagarman et al., 2013). In other words, decreased DNA methylation causes a global shift in the balance of PRC2-mediated methylation towards H3K27me3. Most, if not all, studies to date on the relationship between PRC2 and DNA methylation have focussed on H3K27me3 alone as the read-out of PRC2 function. However, it is interesting to note that DNMT3A-mediated DNA methylation at intergenic chromatin co-exists with the H3K27me2 modification, as well as with H3K36me2 (Fig. 2) (Wu et al., 2010; Weinberg et al., 2019). Therefore, to support our model of molecular interplay, future studies should explore whether loss of DNMT3A function impairs the deposition of intergenic H3K36me2 or H3K27me2. Likewise, DNA methylation profiles should be carefully examined under conditions of altered H3K36me2 and/or H3K27me2 deposition. Supporting this, it has been reported that *NSD1*^+/−^ Sotos syndrome patients exhibit a specific and reproducible DNA methylation signature (Choufani et al., 2015; Weinberg et al., 2019). Remarkably, the specificity of this DNA methylation signature was such that a molecular distinction could be drawn between Sotos syndrome patients and Weaver syndrome patients, despite their highly overlapping clinical phenotypes. Importantly, this finding indicates that a germline mutation in a histone methyltransferase can have specific consequences on DNA methylation patterns that persist and are detectable in adult tissues.

### Weaver syndrome is caused by heterozygous germline mutations in EZH2, SUZ12 and EED

Weaver syndrome (MIM #277590) is an autosomal dominant condition caused by germline monoallelic mutations affecting the genes encoding the core PRC2 subunits, *EZH2*, *EED* and *SUZ12* (Cohen et al., 2015; Gibson et al., 2012; Imagawa et al., 2017; Tatton-Brown et al., 2011). The clinical presentation of Weaver syndrome is characterised chiefly by pre- and postnatal overgrowth, mild-to-moderate intellectual disability, advanced bone age and characteristic craniofacial features, including macrocephaly, round face, broad forehead, hypertelorism, large ears, prominent chin, long philtrum, low nasal bridge and retrognathia (Tatton-Brown and Rahman, 2013).

Although simple haploinsufficiency is not thought to be the mutational mechanism underlying Weaver syndrome, there is a report of an individual who is haploinsufficient for *EZH2* and exhibits some of the features of Weaver syndrome, including overgrowth and intellectual disability (Suri and Dixit, 2017). The overgrowth-associated pathogenic variants of *EZH2*, *EED* and *SUZ12* have, to date, been predicted to be caused by predominantly loss-of-function mutations (Cohen et al., 2016; Imagawa et al., 2017; Lui et al., 2018; Spellicy et al., 2019; Tatton-Brown et al., 2013). However, change- or gain-of-function mutations cannot be ruled out in the absence of more thorough biochemical characterisation.

EZH2 was the first PRC2 subunit to be implicated in Weaver syndrome, with a predominance of missense mutations reported throughout the *EZH2* gene (Gibson et al., 2012). The majority of these missense mutations occur in the catalytic SET domain of EZH2, but some fall within its CXC and SANT domains (Tatton-Brown et al., 2013). Rare truncating mutations in *EZH2* have also been reported, but they all fall within its last exon and therefore likely allow the transcript to escape nonsense-mediated decay (Hentze and Kulozik, 1999). Indeed, such a case was recently reported and it was shown that global EZH2 protein levels in the respective Weaver syndrome patient sample were comparable to those in control samples (Imagawa et al., 2017). As mentioned above, it is difficult to classify these pathogenic *EZH2* variants as being loss-of-, change-in- or gain-of-function mutations because functional characterisation is limited. To date, three studies have attempted to address this by examining the enzymatic activities of Weaver syndrome-associated EZH2 mutant proteins (Cohen et al., 2016; Imagawa et al., 2017; Lui et al., 2018). Collectively, they determined that Weaver syndrome mutant EZH2 exhibits impaired global histone methyltransferase activity both *in vitro* and *in vivo*. The two initial studies did not delineate between the effects on PRC2-mediated H3K27me1, H3K27me2 or H3K27me3 (Cohen et al., 2016; Imagawa et al., 2017). The third and most recent study involved the first described mouse model of Weaver syndrome (Lui et al., 2018). In this study, CRISPR-mediated genome editing was used to generate mice harbouring a Weaver syndrome patient-specific missense mutation targeting the SET domain of EZH2 (p.Val262Met). Mice homozygous for this mutation exhibit perinatal lethality, which contrasts with the early embryonic lethal phenotype of homozygous *Ezh2* loss-of-function mice (Table 1). This observation argues against complete loss of function of the Weaver syndrome mutant EZH2. Mice heterozygous for the *Ezh2*(p.V262M) mutation are viable and born at the expected Mendelian ratio, whereas previously reported heterozygous *Ezh2* loss-of-function mice were not born at the expected Mendelian ratio, but exhibited fewer than expected live births (O'Carroll et al., 2001). Again, the implication is that the Weaver syndrome *Ezh2*(p.V262M) mutant manifests a milder phenotype than complete *Ezh2* loss of function. Nevertheless, both homozygous and heterozygous *Ezh2*(p.V262M) mutant mice exhibit globally decreased H3K27me2 and H3K27me3 (note that H3K27me1 levels were not examined). Taken together, these data support a partial loss-of-function phenotype for the Weaver syndrome mutant EZH2. Importantly, heterozygous *Ezh2*(p.V262M) mice also exhibit postnatal overgrowth, a key feature of Weaver syndrome.

The reported mutations in *EED* and *SUZ12* are also predicted to be loss-of-function mutations but, so far, lack thorough biochemical characterisation (Cohen et al., 2015; Imagawa et al., 2017). EED is recurrently affected by nonsynonymous mutations at a few key residues within its WD-40 domain, a key structural feature of the EED protein required for recognition of H3K27me3 on chromatin and allosteric activation of PRC2 (Oksuz et al., 2018; Spellicy et al., 2019). Likewise, two of three reported Weaver syndrome-associated mutations in *SUZ12* are nonsynonymous substitutions within its VEFS domain, a key functional domain for its association with EZH2 and EED, which, when deleted, abolishes the enzymatic activity of PRC2 (Højfeldt et al., 2018; Imagawa et al., 2018). In summary, although the genetic aetiology of Weaver syndrome can be variable, PRC2 dysfunction clearly plays a central role in the developmental origins of the disease.

### Monoallelic loss-of-function mutations in NSD1 cause Sotos syndrome

Sotos syndrome (MIM #117550) is an autosomal dominant developmental disorder resulting from either germline haploinsufficiency of, or intragenic loss-of-function mutations in *NSD1* (Tatton-Brown and Rahman, 2013). At the phenotypic level, Sotos syndrome is remarkably similar to Weaver syndrome, characterised primarily by pre- and postnatal overgrowth, mild-to-severe intellectual disability, advanced bone age and characteristic craniofacial features that include downward slanting palpebral fissures, a long and thin face, and a prominent chin and broad forehead.

The mutational spectrum of Sotos syndrome is diverse, including nonsense and missense mutations, partial and whole-gene deletions, intragenic indels and splice-site mutations. However, it appears clear that pathogenic variants always abrogate NSD1 function (Tatton-Brown et al., 2005). For example, pathogenic missense mutations in *NSD1* occur exclusively within functional domains of the protein that are implicated in chromatin regulation (Tatton-Brown et al., 2005). Furthermore, *in vitro* assays have determined that the H36 methyltransferase activity of NSD1 is impaired in Sotos syndrome-mutant versions of the enzyme (Qiao et al., 2011). Interestingly, loss-of-function mutations in the related H3K36 histone methyltransferase SETD2 cause a similar but less severe ‘Sotos-like’ syndrome. This observation is resonant with what is seen in homozygous loss-of-function mouse models, with *Setd2*-null embryos exhibiting a less severe developmental mutant phenotype than *Nsd1*-null embryos (Table 1) (Hu et al., 2010; Rayasam et al., 2003).

### *De novo* DNMT3A mutations are implicated in Tatton-Brown–Rahman syndrome

Tatton-Brown–Rahman syndrome (MIM #615879) is an autosomal dominant genetic condition resulting from germline heterozygous mutations in the *DNMT3A* gene (Okamoto et al., 2016). The key clinical features of Tatton-Brown–Rahman syndrome are reminiscent of those observed in Weaver and Sotos syndromes, and include tall stature, mild-to-moderate intellectual disability and distinctive craniofacial characteristics, including macrocephaly, a round face, heavy horizontal eyebrows and narrow palpebral fissures (Okamoto et al., 2016). Tatton-Brown–Rahman syndrome has only relatively recently been defined at the clinical level and so its molecular characterisation is yet lacking.

The spectrum of reported pathogenic mutations in *DNMT3A* includes microdeletions, in-frame deletions, frameshift insertions and missense mutations (Tatton-Brown et al., 2014). Although the biochemical and functional characterisation of these mutations remain to be determined, they are predicted to interfere with the intra- and inter-molecular protein-protein interactions of DNMT3A and ultimately disrupt its ability to methylate DNA accurately (Tatton-Brown et al., 2014). Given that haploinsufficiency of *DNMT3A* has been ruled in as a mutational mechanism for Tatton-Brown–Rahman syndrome, it seems likely that all pathogenic variants will prove to be loss of function (Okamoto et al., 2016).

### Imbalanced regulation of PRC2 at intergenic chromatin as a common feature of overgrowth syndromes

Considering the convergence of PRC2-mediated H3K27me2, NSD1-mediated H3K36me2 and DNMT3A-mediated DNA methylation at intergenic chromatin, we propose that an equilibrium exists between these modifications and that a shared molecular feature of the abovenamed developmental disorders may be disruptions to this balance, which shift the landscape of PRC2-mediated methylation (Fig. 2). Supporting this, there is evidence for gene dosage effects of *NSD1* and *DNMT3A* on human growth (Table 2). In other words, an increase in the copy number of either gene appears to have the opposite effect on growth compared with having one loss-of-function mutation in that same gene. For example, whereas *NSD1* haploinsufficiency is associated with overgrowth, reciprocal duplications involving *NSD1* correlate with opposing clinical features, including growth retardation, delayed bone age and microcephaly (Dikow et al., 2013; Rosenfeld et al., 2013). Similarly, an individual with a maternally inherited duplication encompassing *DNMT3A* exhibits a growth failure phenotype marked by developmental delay, despite also possessing a paternally inherited Weaver syndrome-related point mutation in *EZH2* (Polonis et al., 2018). This phenotype suggests that the increased gene dosage of *DNMT3A* exerts dominant effects that serve to restrict growth, masking any contribution from the missense *EZH2* mutation, which normally causes overgrowth. It should be noted, however, that for both of the above cases, a functional increase in the activity of the duplicated gene product is yet to be confirmed.

More complex, change-of-function mutations affecting the PWWP domain of *DNMT3A*, which abrogate its ability to bind to H3K36me2 and H3K36me3 *in vitro* but result in DNA hypermethylation at sites marked by H3K27me3, also result in growth restriction in both mice and humans (Heyn et al., 2018; Sendžikaitė et al., 2019). Although only *DNMT3A* has been mutated in this context, the mutation has functional consequences on the normal crosstalk with NSD1-mediated H3K36me2, and an imbalance in the landscape of DNA methylation specifically at PRC2 target sites is observed. This result is consistent with our model in which a carefully balanced equilibrium exists between PRC2, NSD1 and DNMT3A that, when skewed, can affect growth regulation. Supporting this, missense mutations affecting the PWWP domain of DNMT3A are also reported in Tatton-Brown–Rahman syndrome which similarly abrogate its ability to bind H3K36me2 and H3K36me3 *in vitro*, but contrastingly manifest overgrowth and DNA hypomethylation similar to that observed in Sotos syndrome (Weinberg et al., 2019). Our model would predict that the respective PWWP domain mutations in DNMT3A differ in their downstream effects on PRC2-mediated H3K27 methylation.

A Weaver syndrome-associated mutation affecting the H3K36-sensing pocket in EZH2 is also reported to disrupt the crosstalk between PRC2 and NSD1 on chromatin (Jani et al., 2019). When compared with wild-type PRC2 in *in vitro* assays, disease-associated mutant EZH2-containing PRC2 exhibits increased enzymatic activity on H3K36-trimethylated nucleosomes, despite exhibiting decreased overall HMT activity on unmodified nucleosomes (Jani et al., 2019). This change would be predicted to shift the balance between H3K27me2 and H3K36me2 at intergenic chromatin, as well as potentially disrupting the profiles of H3K27 and H3K36 methylation at a genome-wide level. Again, this result is consistent with a model of disturbed crosstalk between PRC2 and NSD1 as a feature of Weaver syndrome.

Clearly, changes in the crosstalk between PRC2, NSD1 and DNMT3A that affect the balance of their associated modifications can have consequences on human growth. We propose that imbalances at intergenic chromatin are particularly relevant to the aetiology of the Weaver, Sotos and Tatton-Brown–Rahman syndromes. Supporting this, it has been shown that NSD-mediated H3K36me2 is specifically required for intergenic DNMT3A localisation and DNA methylation (Weinberg et al., 2019). Furthermore, although the functions of DNMT3A and DNMT3B largely overlap, DNMT3B differs in that it preferentially methylates genic DNA (Baubec et al., 2015; Weinberg et al., 2019). Accordingly, mutations in the *DNMT3B* gene do not cause human overgrowth, indicating that disruptions to the non-overlapping functions of DNMT3A (i.e. non-genic DNA methylation) are what contribute to the overgrowth phenotype. Similarly, SETD2 tri-methylates H3K36 at active gene bodies, but heterozygous loss-of-function mutations in *SETD2* causes a less severe ‘Sotos-like’ phenotype (Table 2). This might suggest a less important role for genic H3K36 methylation in the regulation of growth and development (Luscan et al., 2014; Tlemsani et al., 2016). Furthermore, genes encoding members of cPRC1 and ncPRC1, which colocalise with PRC2-mediated H3K27me3 at unmethylated CpG islands, have so far not been reported to be mutated in human overgrowth syndromes. In fact, a mutation in their core component, *RING1A*, causes a dissimilar, neurodevelopmental disorder (Pierce et al., 2018). Taken together, these findings suggest that imbalances in chromatin modifications at gene bodies and CpG islands are not foremost in the pathogenesis of the human overgrowth syndromes, leaving intergenic chromatin as the key candidate.

## Conclusions

Here, we have proposed a new molecular viewpoint from which the phenotypic overlap between the genetically distinct Weaver, Sotos and Tatton-Brown–Rahman overgrowth syndromes may be understood. We speculate that aberrations in the crosstalk between PRC2, NSD1 and DNMT3A, and an imbalance in their associated modifications at intergenic chromatin, might be a key shared feature of these distinct, but related, developmental disorders. However, experimental testing of this hypothesis will be required. To this end, future studies seeking to characterise the molecular aetiology of Weaver, Sotos or Tatton-Brown–Rahman syndrome could widen their scope to incorporate analyses of H3K27me2, H3K36me2 and DNA methylation. The study of DNA methylation patterns in these disorders is likely to be of particular clinical value. DNA methylation profiling is already in use as a diagnostic tool for various cancers, and it holds promise as a method to discriminate between the clinically overlapping Weaver, Sotos and Tatton-Brown–Rahman syndromes at the molecular level. Future analyses of the enzymatic activities of mutant forms of core PRC2 members should also be refined to delineate between the ability to mediate H3K27me3, H3K27me2 and H3K27me1, and genomic profiling of cells from patients should be extended to look for alterations in the deposition of these modifications at intergenic chromatin.

To date, mouse models of Weaver, Sotos and Tatton-Brown–Rahman syndrome are limited. Assuming loss of function as the primary mutational mechanism of disease, it is striking that no growth-related phenotypes have been reported for heterozygous loss-of-function *Ezh2*, *Eed*, *Suz12*, *Nsd1* or *Dnmt3a* mutant mice (Table 1). This may simply be a reporting issue, as developmental overgrowth phenotypes can be relatively mild in mice and therefore can easily go unnoticed by a researcher who is more focussed on the homozygous condition and/or is not explicitly searching for subtle growth-associated phenotypes. Another possibility is that the assumption of a simple loss-of-function mutational mechanism may be incorrect. Alternative mechanisms – such as dominant-negative, or cooperative gain-of-function effects – may be investigated by generating mouse models harbouring patient-specific mutations, as has been done for Weaver syndrome (Lui et al., 2018). However, even such purpose-engineered mice may fail to phenocopy all the key elements of a given human overgrowth syndrome. It might simply not be possible to recapitulate fully the pathophysiology of human overgrowth syndromes in a mouse model; given the developmental origins of these syndromes, perhaps gestation period – which is much shorter in mice – plays an important role in contributing to the severity of the mutant phenotype.

An alternative and promising experimental avenue for characterising overgrowth-associated mutations lies in the derivation of induced pluripotent stem cells (iPSCs) from patients. With the advent of CRISPR-Cas9 genome editing, isogenic control cell lines could be generated by replacing or repressing the mutant allele. Such an approach would have the added benefit of helping to define the molecular nature of the mutations in Weaver syndrome: if repression of the mutant gene product alone can restore the wild-type phenotype, then simple loss of function or haploinsufficiency can be ruled out definitively and change-in- or gain-of-function mutations with dominant effects ruled in as the mutational mechanism. Furthermore, by employing an iPSC model, experiments can be performed under differentiation conditions in order to get a clearer picture of what might be going awry during the developmental process to ultimately produce the overgrowth syndrome phenotype.

Although many open questions remain regarding the chromatin crosstalk between PRC2, NSD1 and DNMT3A, the molecular tools are already in place to start addressing them. Importantly, our ever-increasing understanding of their normal structure, function and interplay on chromatin and during development will continue to shed light on the potential mechanisms underlying the remarkable phenotypic overlap of the Weaver, Sotos and Tatton-Brown–Rahman overgrowth syndromes.
